# PG102 Upregulates IL-37 through p38, ERK, and Smad3 Pathways in HaCaT Keratinocytes

**DOI:** 10.1155/2019/6085801

**Published:** 2019-02-24

**Authors:** Hyun-keun Kim, Seonung Lim, Min-Jung Bae, Wonwoo Lee, Sunyoung Kim

**Affiliations:** ^1^School of Biological Sciences, Seoul National University, Seoul 151-742, Republic of Korea; ^2^ViroMed Co. Ltd., Building 203, Seoul National University, Seoul 151-742, Republic of Korea

## Abstract

IL-37 is an immunomodulatory cytokine that suppresses inflammation in various cell types and disease models. However, its role in keratinocytes has not been clearly understood, and there has been no report on the agents that can increase the expression of IL-37 in keratinocytes. In this study, we investigated the effects of silencing *IL37* in HaCaT keratinocytes and the molecular mechanisms involved in the upregulation of IL-37 by PG102, a water-soluble extract from *Actinidia arguta*. It was found that knockdown of *IL37* resulted in the augmented expression of antimicrobial peptides (AMPs) in response to cytokine stimulation. PG102 increased the expression of IL-37 at both mRNA and protein levels presumably by enhancing the phosphorylation of Smad3, ERK, and p38. Indeed, when cells were treated with specific inhibitors for these signaling molecules, the expression level of IL-37 was reduced. PG102 also promoted colocalization of phospho-Smad3 and IL-37. Our results suggest that IL-37 inhibits the expression of AMPs and that PG102 upregulates IL-37 through p38, ERK, and Smad3 pathways in HaCaT cells.

## 1. Introduction

Interleukin 37 (IL-37) is a member of the IL-1 cytokine family whose function as a fundamental inhibitor of innate immunity was first discovered in 2010 [1]. Unlike other IL-1 family members such as IL-1*β*, IL-18, and IL-36, which are proinflammatory, IL-37 exhibits anti-inflammatory properties in various cell types [1–3]. Although the mouse ortholog of IL-37 has not been discovered, delivery of the human IL-37 gene to mice showed dampened inflammatory responses in animal models of lipopolysaccharide- (LPS-) induced shock, inflammatory bowel disease, and insulin resistance [1, 4, 5].

IL-37 can be induced by proinflammatory mediators such as IL-1*β*, tumor necrosis factor- (TNF-) *α*, toll-like receptor (TLR) agonists such as LPS, and growth factors such as epidermal growth factor (EGF) and transforming growth factor beta (TGF-*β*) [1, 6]. Its expression and activation require cleavage by caspase-1, which induces both secretion and nuclear translocation of the IL-37 protein [7]. When acting intracellularly, IL-37 forms a functional complex with phosphorylated Smad3 and translocates into the nucleus [1, 8]. On the other hand, inhibition of Smad3 not only suppresses the expression of IL-37 but also reverses the inhibition of proinflammatory cytokines in macrophage and lung epithelial cell lines [1]. Thus, Smad3 activities are essential for IL-37 to exert its anti-inflammatory effects.

Psoriasis is an inflammatory skin disease characterized by the abnormal differentiation and hyperproliferation of keratinocytes [9]. Keratinocytes in psoriatic epidermis are constitutively stimulated by cytokines like IL-17A and IL-22, and they in turn secrete various antimicrobial peptides (AMPs) that are known to amplify the disease [10]. While these factors are overexpressed in psoriatic lesions, transcriptome analysis of psoriatic lesional skin has revealed that *IL37* was one of the most downregulated genes compared to healthy skin [11]. In addition, the overexpression of IL-37 in HaCaT keratinocytes suppressed the production of proinflammatory cytokines, and the delivery of plasmid encoding IL-37 into keratin 14-VEGF transgenic mice ameliorated the symptoms of psoriasis [12]. Thus, the upregulation of IL-37 in the skin may be an effective therapeutic approach to alleviate inflammatory skin diseases.

PG102 is a standardized extract from an edible portion of *Actinidia arguta*. It has been shown to alleviate clinical symptoms of various animal disease models including spontaneous dermatitis, atopic dermatitis, and psoriasis-like skin inflammation [13–15]. Furthermore, an exploratory human clinical study with 90 asymptomatic atopy patients (serum total IgE > 300 IU/mL) has verified significant immunomodulatory effects along with the safety of PG102 [16]. Recently, we have reported that PG102 suppresses the expression of AMPs in HaCaT cells stimulated with inflammatory cytokines [15].

To date, there is no published paper reporting an agent that can upregulate IL-37 in keratinocytes, and the role of IL-37 in keratinocytes has remained elusive. Here, we demonstrated that silencing *IL37* leads to increased inflammatory responses in HaCaT cells and that PG102 upregulated IL-37 levels through extracellular signal-related kinases (ERK)/mothers against decapentaplegic homolog 3 (Smad3) and p38 while promoting the colocalization of IL-37 and phospho-Smad3. These results suggest potential anti-inflammatory roles of PG102 through the regulation of IL-37 expression and possible application of PG102 against inflammatory skin diseases.

## 2. Materials and Methods

### 2.1. Reagents

PG102 was prepared, and its batch-to-batch consistency was controlled as previously described [15, 17, 18]. Briefly, the dried fruit of *Actinidia arguta* was extracted in boiling water for 3 hours, followed by filtration, concentration, and lyophilization. Quality control was performed by measuring the chemical contents of marker compounds and IL-8 bioassay in HaCaT cells. Recombinant IL-1*α*, IL-17A, IL-22, Oncostatin M, and TNF-*α* were purchased from BioLegend (San Diego, CA). ERK inhibitor U0126, p38 inhibitor SB203580, and Smad3 inhibitor SIS3 were obtained from Selleckchem (Houston, TX). Chemical inhibitor stocks were prepared at 50 mM. For all of the experiments, the concentrations of DMSO in the cell cultures were lower than 0.1%.

### 2.2. Cell Culture and siRNA Transfection

Human keratinocyte cell line HaCaT was purchased from CLS Cell Lines Service GmbH (Eppelheim, Germany). Cells were serially passaged at 70~80% confluence in Dulbecco's modified Eagle's medium (DMEM; Thermo Fisher, Waltham, MA) containing 10% fetal bovine serum (FBS; Corning, Corning, NY) and antibiotics (100 U/mL penicillin and 100 mg/mL streptomycin) at 37°C in a 5% CO_2_ humidified incubator. Cells at passage 3 to 5 were used throughout the experiment.

For siRNA-mediated knockdown of *IL37*, 5 × 10^4^ cells (*n* = 3) were seeded onto a 12-well plate overnight. After replacement of the culture medium, Silencer Select control siRNA and *IL37* siRNA (Invitrogen, Waltham, MA) were added with RNAiMAX transfection reagent (Invitrogen, Waltham, MA), followed by 48 hours of incubation. Cells were then washed once with PBS and incubated with cytokines for an additional 24 hours for further analysis.

### 2.3. Total RNA Extraction and Real-Time Quantitative PCR (RT-qPCR)

2 × 10^5^ cells/mL HaCaT cells were seeded onto 12-well cell culture plates overnight (*n* = 3) and treated with PG102 at designated concentrations at different time points. Treatment with PG102 at 0.25~2.0 mg/mL did not cause cytotoxicity in HaCaT cells [15]. Total RNA was isolated from cells using RNAiso (Takara Bio, Shiga, Japan) according to the manufacturer's protocol, followed by complementary DNA (cDNA) synthesis using AMV reverse transcriptase (Takara Bio) and oligo dT primers (Qiagen, Valencia, CA). Real-time quantitative PCR (RT-qPCR) of each cDNA was performed using SYBR Premix Ex Taq™ (Takara Bio) and Thermal Cycler Dice Real Time System (Takara Bio) with the primer pairs listed in Table 1. The mRNA levels were normalized by the level of HPRT, and the relative changes in gene expression to untreated controls were calculated by the 2-^ΔΔCt^ method.

### 2.4. Western Blot Analysis

HaCaT cells were seeded at a density of 4.5 × 10^5^ cells/well onto 6-well plates overnight. Cells were then treated with PG102 at designated concentrations for 48 hours, and total cell lysates were extracted with CytoBuster™ (Merck, Darmstadt, Germany) mixed with PhosSTOP™ and cOmplete™ Protease Inhibitor Cocktail (Roche, Basel, Switzerland). Total protein contents were measured by the BCA assay kit (Invitrogen), and after reconstitution in a sample buffer, 20 micrograms of protein samples was subjected to SDS-PAGE on Bolt™ 10% Bis-Tris Plus Gels (Invitrogen). Proteins were transferred onto a PVDF membrane, and the membrane was incubated in 5% skim milk in 0.1% TBST at room temperature for 1 hour to block nonspecific binding. The membrane was then incubated with antibodies specific to IL-37, phopho-Smad3 (1 : 1000; Abcam), Smad3, phospho-ERK, ERK (1 : 1000; Cell Signaling Technology, Danvers, MA), and *β*-actin (1 : 5000; Sigma-Aldrich) overnight at 4°C followed by incubation with a horseradish peroxidase-conjugated secondary anti-mouse or anti-rabbit IgG (1 : 100,000; Sigma-Aldrich) at room temperature for 1 hour. The blot was developed by Immobilon ECL HRP substrate (Merck) and visualized by exposure to autoradiography film.

### 2.5. Immunofluorescence Staining

HaCaT cells were seeded in 4-well Lab-Tek II Chamber Sliders (Nunc, Rochester, NY) at a density of 5 × 10^4^ cells/well overnight. Thereafter, cells were incubated with PG102 for designated time points, followed by cold phosphate-buffered saline (PBS) wash and fixation with 4% paraformaldehyde. Each slide was blocked in 5% donkey serum and 10% FBS for 1 hour at room temperature. The slides were then incubated with antibodies specific to IL-37 (1 : 200, Thermo Fisher) and phospho-Smad3 (1 : 200, Abcam) overnight at 4°C. This was followed by 1 hour incubation with the respective secondary antibodies and mounted with Vectashield 4′,6-diamidino-2-phenylindole (DAPI) mounting medium. Digital confocal imaging was performed and analyzed using Carl Zeiss (Oberkochen, Germany) LSM 700 confocal microscope and ZEN software.

### 2.6. Statistical Analysis

The data are presented as the mean ± standard deviation (SD) of triplicate measurements, and each experiment was repeated at least three times. Statistical analysis was performed using the GraphPad Prism version 6.0 (GraphPad Software, San Diego, CA). Comparisons with other experimental groups were analyzed by one-way analysis of variance (ANOVA) followed by the Bonferroni post hoc test. *P* values less than 0.05 were considered statistically significant.

## 3. Results

### 3.1. Silencing IL-37 Increases the Expression of Antimicrobial Peptides in HaCaT Cells

**Figure 1:**
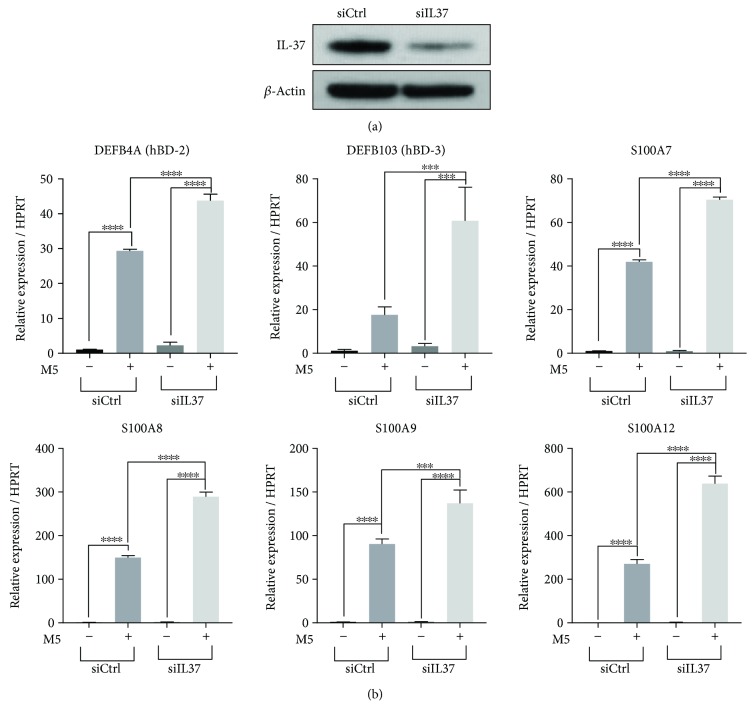
Silencing of *IL37* augments inflammatory responses to cytokines in HaCaT cells. (a) siRNA-mediated knockdown of *IL37* was confirmed by Western blot analysis. (b) HaCaT cells were transfected with the control and *IL37* siRNAs and treated with the mixture of five cytokines (M5) for 24 hours, followed by RT-qPCR analysis. Representative results from three independent experiments are shown. Each point represents mean ± SD. ^∗∗^*P* < 0.01, ^∗∗∗^*P* < 0.001, ^∗∗∗∗^*P* < 0.0001.

IL-37 is a suppressor of proinflammatory responses induced by inflammatory insults such as LPS or cytokines [1]. The effects of silencing endogenous IL-37 have been reported in human peripheral blood mononuclear cells (PBMCs), aortic valve interstitial cells, and renal tubular epithelial cells but have never been tested in the context of keratinocytes [7, 19–21]. We initially tested the effects of *IL37* knockdown by siRNA in the human keratinocyte cell line, HaCaT cells (Figure 1(a)). After silencing *IL37*, cells were stimulated with a mixture of five cytokines designated as M5 (IL-1*α*, IL-17A, IL-22, Oncostatin M, and TNF-*α*) to mimic the microenvironment of keratinocytes in psoriasis [22]. In cells transfected with control siRNA, treatment with M5 resulted in increased expressions of various AMPs including *DEFB4A* (hBD-2), *DEFB103* (hBD-3), *S100A7*, *S100A8*, *S100A9*, and *S100A12* (Figure 1(b)). These effects were further augmented by transfection of *IL37* siRNA, suggesting that IL-37 acts as a natural inhibitor of inflammation in HaCaT cells, as in the case of other cell types (Figure 1(b)). Based on this result, it was hypothesized that upregulating IL-37 may be an effective approach to regulate excessive inflammation in keratinocytes.

### 3.2. PG102 Upregulates IL-37 mRNA and Protein Levels in HaCaT Cells

**Figure 2:**
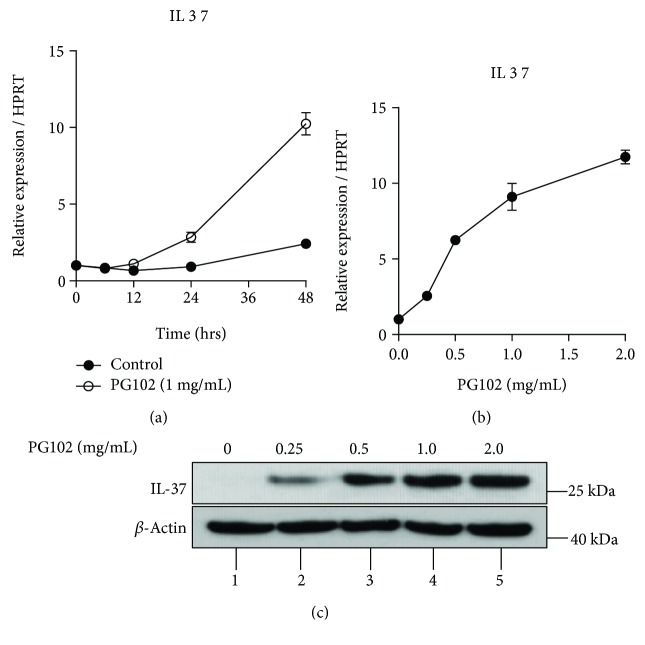
PG102 upregulates IL-37 expression in HaCaT cells. (a) PG102 (1 mg/mL) was treated to HaCaT cells at different time points, followed by RNA extraction and RT-qPCR analysis. (b) HaCaT cells were treated with PG102 at different concentrations for 24 hours, followed by RT-qPCR analysis. (c) HaCaT cells were treated with PG102 at different concentrations for 48 hours, followed by Western blot analysis. Representative results from three independent experiments are shown. Each point represents mean ± SD. ^∗∗∗^*P* < 0.001, ^∗∗∗∗^*P* < 0.0001 versus control cells.

We have previously shown that PG102 represses the expression of AMPs such as hBD-2 and S100A8/A9, which are highly increased upon exposure to inflammatory cytokines in HaCaT cells [15]. Based on the anti-inflammatory properties of PG102, it was tested whether PG102 might also enhance the expression of IL-37. When HaCaT cells were treated with PG102, the mRNA level of *IL37* started to increase from 24 hours after treatment and continued to the last time point, 48 hours (Figure 2(a)). Treatment with PG102 at different concentrations induced *IL37* expression in a dose-dependent manner (Figure 2(b)). At 2 mg/mL of PG102, the mRNA level of *IL37* increased more than 10-fold compared to the control group. The protein expression of IL-37 was also increased by PG102 (Figure 2(c)). These results suggest that PG102 upregulates IL-37 expression at both mRNA and protein levels in HaCaT cells.

### 3.3. Activation of Smad3 Is Essential for the Induction of IL-37 by PG102 in HaCaT Cells

**Figure 3:**
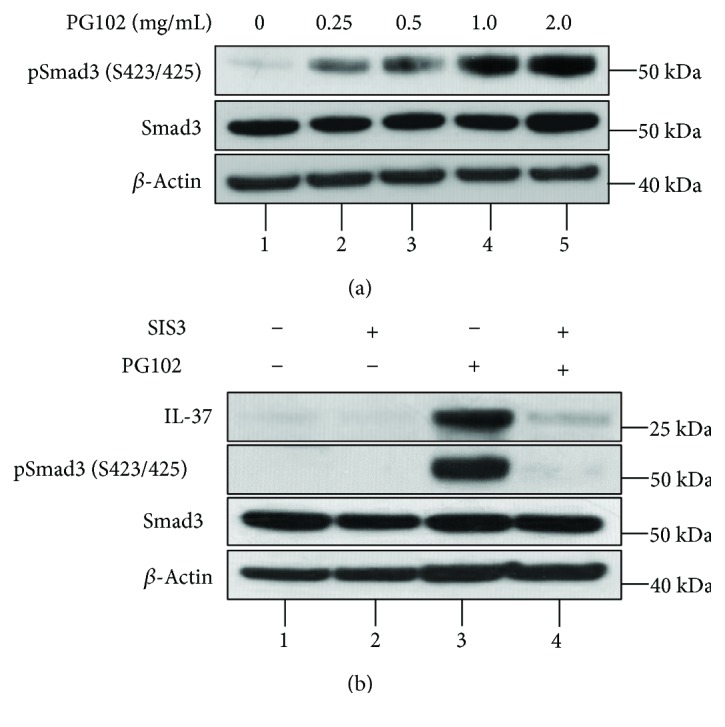
IL-37 induction by PG102 is dependent on the activation of Smad3. (a) HaCaT cells were incubated with PG102 for 48 hours. The cells were lysed, and equal amounts of protein were subjected to Western blot analysis using antibodies against phospho-Smad3, Smad3, and *β*-actin. (b) HaCaT cells were pretreated with 10 *μ*M SIS3 for 1 hour, followed by incubation with or without PG102 (1 mg/mL) for 48 hours and Western blot analysis. Representative results from three independent experiments are shown.

It has previously been shown that activation of Smad3 is essential for the expression of IL-37 [1]. Therefore, it was tested whether Smad3 phosphorylation was involved in the PG102-mediated induction of IL-37. As shown in Figure 3(a), HaCaT cells treated with PG102 showed an increase in the phosphorylation of Smad3 in a concentration-dependent manner. Thus, it was predicted that PG102 could enhance IL-37 expression by activating Smad3. To confirm this, SIS3—a specific inhibitor of Smad3—was used to block the activation of Smad3. Cells were pretreated with SIS3 for two hours followed by a treatment with PG102. As shown in Figure 3(b), PG102-mediated expression of IL-37 was effectively suppressed by pretreatment with SIS3. These results show that Smad3 is necessary for the induction of IL-37 by PG102.

### 3.4. PG102 Increases IL-37 Expression through Phosphorylation of ERK and p38 MAPKs in HaCaT Cells

**Figure 4:**
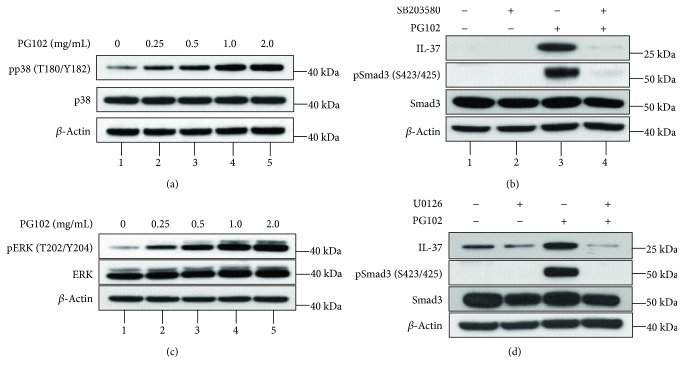
PG102 increases IL-37 expression through p38 and ERK/Smad3 axis. (a) HaCaT cells were treated with PG102 at different concentrations for 30 minutes, and the protein levels of phospho-p38 and p38 were analyzed by Western blot analysis. (b) Cells were pretreated with SB203580 (20 *μ*M) for 1 hour, followed by treatment with PG102 (1 mg/mL) for 48 hours. The protein levels of IL-37, phospho-Smad3, and Smad3 were analyzed by Western blot analysis. (c) Cells were treated with PG102 at different concentrations for 30 minutes, and the protein levels of phospho-ERK1/2 and ERK1/2 were analyzed by Western blot analysis. (d) Cells were pretreated with U0126 (5 *μ*M) for 1 hour, followed by treatment with PG102 (1 mg/mL) for 48 hours. The protein levels of IL-37, phospho-Smad3, and Smad3 were analyzed by Western blot analysis. Representative results from three independent experiments are shown.

Activation of mitogen-activated protein kinases (MAPKs) leads to phosphorylation of a variety of their downstream targets including Smad3 and promotes transcription of various genes [23]. Since MAPKs have been shown to be required for the induction of IL-37, we investigated the role of MAPKs in the PG102-mediated upregulation of IL-37. Treatment with PG102 enhanced the phosphorylation of p38 and ERK in a concentration-dependent manner (Figures 4(a) and 4(c)).

Next, it was tested whether inhibiting p38 and ERK activities reverses the effects of PG102 on Smad3 phosphorylation and IL-37 expression. Cells were incubated in the presence of SB203580 and U0126—specific inhibitors of p38 and ERK, respectively—for 1 hour prior to treatment with PG102. As shown in Figure 4(b), inhibition of p38 reduced the expression of the IL-37 and phosphorylated Smad3. In addition, pretreatment with U0126 diminished both phosphorylated and total forms of Smad3, as well as expression of IL-37, suggesting that both p38 and ERK are the upstream kinases responsible for phosphorylating Smad3 and inducing IL-37 (Figure 4(d)). Taken together, these results show that p38, ERK, and Smad3 pathways are involved in the PG102-mediated induction of IL-37.

### 3.5. PG102 Promotes Colocalization of IL-37 and Smad3 in Perinuclear Regions in HaCaT Cells

**Figure 5:**
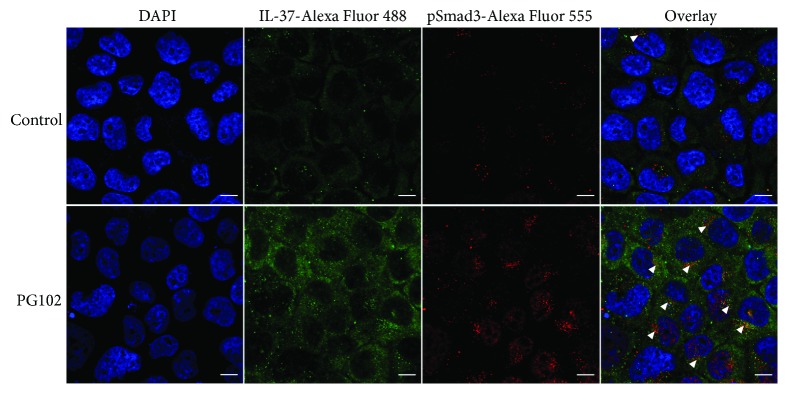
PG102 promotes colocalization of IL-37 and phospho-Smad3. Confocal microscopy of HaCaT cells treated with PG102 (1 mg/mL) for 24 hours. Localization of nuclei (DAPI), IL-37 (Alexa Fluor 488), and pSmad3 (Alexa Fluor 555) is shown. Colocalized proteins are shown in white arrowheads. Scale bar: 10 *μ*m.

IL-37 can bind to intracellular Smad3 to form a functional complex in the perinuclear regions, ultimately translocating to the nucleus and controlling transcription of a wide variety of inflammatory genes [1]. Using immunofluorescence, we assessed whether PG102 could promote colocalization of IL-37 and Smad3. As observed in Figure 5, IL-37 and phospho-Smad3 were induced by PG102, and they were costained in the perinuclear regions. These results suggest that PG102 not only increases the expression of IL-37 but also induces the formation of a functional complex of IL-37 and Smad3.

## 4. Discussion

IL-37 is a potent immunomodulatory cytokine that exerts a broad range of biological functions. Silencing IL-37 results in excessive inflammatory responses to external stimuli in various cell types, but little is known about the role of IL-37 in keratinocytes [24]. Here, we demonstrated that knockdown of *IL37* augmented the expression of AMPs in cytokine-stimulated HaCaT keratinocytes. Based on this result, we speculated that upregulation of IL-37 in HaCaT cells might be an effective approach to suppress excessive inflammation. Since PG102, a standardized water-soluble extract from *Actinidia arguta*, has previously been shown to inhibit the expression of various AMPs in HaCaT cells, it was investigated whether IL-37 was involved in the biological effects of this botanical extract [15]. In this study, we showed that PG102 could significantly increase IL-37 levels through the control of p38, ERK, and Smad3 pathways.

In the cytokine milieu of psoriatic skin, keratinocytes release a variety of AMPs such as S100 proteins and beta defensins. Although AMPs were initially known as simple peptides with antimicrobial functions, it is now widely accepted that they actively participate in immune responses [25]. For instance, S100A8 and S100A9 form a complex called calprotectin that aggravates psoriasis by regulating expression of the C3 complement factor, while hBD-2 promotes chemotaxis of various leukocytes in a C-C chemokine receptor (CCR) 2- and CCR6-dependent manner [26, 27]. Consistent with these results, transcriptome analyses of psoriatic lesions have identified AMPs as one of the most upregulated groups of genes [28, 29]. On the other hand, *IL37* was shown to be one of the most downregulated genes in psoriatic lesions [11]. These results raise the possibility that the chronic inflammation in psoriasis may be due, in part, to the reduced expression of anti-inflammatory mediators like IL-37. This is supported by another study that has shown that overexpression of IL-37 in HaCaT cells effectively mitigated production of IL-6, IL-8, and S100A7 [12]. Therefore, induction of IL-37 in keratinocytes by PG102 may be a novel approach to alleviate psoriasis.

To study the molecular mechanism underlying PG102-mediated upregulation of IL-37, we investigated the effects of PG102 on Smad3 and MAPKs. One study has indicated that hBD-3 increases the expression of IL-37 in human keratinocytes through the use of such molecules as Smad3, ERK1/2, c-Jun N-terminal kinases (JNK), and nuclear factor-*κ*B (NF-*κ*B) [6]. PG102 upregulated IL-37 by activating Smad3, ERK1/2, and p38 but not involving JNK. We had previously shown that PG102 suppresses NF-*κ*B signaling in HaCaT cells [15]. Hence, it is anticipated that the signaling pathways involved in the expression of IL-37 may vary depending on the cell type and stimulants used. Whatever the case is, Smad3 seems to be the key molecule orchestrating IL-37 across different cell types.

IL-37 has dual functions in that it can act both extracellularly and intracellularly [7, 30]. Similar to other IL-1 family cytokines, IL-37 is secreted outside the cells after cleavage by caspase-1 and binds to either IL-18 receptor *α* (IL-18R*α*) or single Ig IL-1–related receptor (SIGIRR) to exert its effects [30, 31]. Inside cells, however, it forms a functional complex with Smad3, which is required for its anti-inflammatory effects [1]. It has been reported that silencing Smad3 diminishes the anti-inflammatory effects of IL-37 in various cell types and IL-37tg mice [1, 32]. These observations are consistent with the well-known function of Smad3, which translocates into the nucleus after being phosphorylated and binds to the Smad-binding element (SBE) present in the promoter of respective genes [33]. For instance, it antagonizes the activation of signal transducer and activator of transcription 3 (STAT3) and the subsequent production of IL-6, which is a major inflammatory molecule involved in psoriasis [34]. Our results suggested that PG102 might control the intracellular function of IL-37 in HaCaT cells as we could not detect IL-37 in cell culture supernatants using ELISA. However, it may also be possible that HaCaT cells may be defective in the machinery involved in the processing of IL-37. It has been reported that HaCaT cells express a relatively low level of caspase-1 compared to primary keratinocytes or THP-1 cells [35]. Thus, to elucidate whether PG102 affects the secretion of IL-37, different cells such as primary keratinocytes should be employed.

It is not yet clear which compounds in PG102 upregulate the expression of IL-37. PG102 contains numerous compounds with anti-inflammatory properties [36]. One particular group of molecules of interest is the glycosides, as these molecules isolated from *Tripterygium wilfordii* Hook F have been shown to possess strong anti-inflammatory properties and upregulate IL-37 in THP-1 human macrophage cell line [37, 38]. Glycosides of various compounds are abundant in PG102 as well, so they can be obvious candidate molecules responsible for the upregulation of IL-37 [39, 40]. Further investigations are underway to identify active compounds from PG102.

## 5. Conclusion

Our findings demonstrate that silencing *IL37* intensifies the expression of AMPs in response to cytokine stimulation, suggesting that IL-37 acts as an inhibitor of inflammation in HaCaT cells. PG102 significantly increased IL-37 expression at both mRNA and protein levels, and these effects were mediated through p38, ERK, and Smad3 pathways. Furthermore, PG102 promoted colocalization of IL-37 and phosphorylated Smad3 in the perinuclear regions, which is essential for the anti-inflammatory activities of IL-37. Currently, there is no report on agents that can induce the expression of IL-37 in keratinocytes. Our results suggest that PG102 might be used as a basis for developing therapeutics for inflammatory skin diseases such as psoriasis.

## Figures and Tables

**Table 1:** List of primers used for RT-qPCR.

Gene	Primer	Sequence (5′-3′)
IL37	F	GGACAAAGTCATCCATCCCTTC
	R	GAGCCCACCTGAGCCCTATAA
HPRT	F	TATGGCGACCCGCAGCCCT
	R	CATCTCGAGCAAGACGTTCAG
DEFB4	F	GGTGTTTTTGGTGGTATAGGC
	R	AGGGCAAAAGACTGGATGACA
DEFB103	F	TGAAGCCTAGCAGCTATGAGGATC
	R	CCGCCTCTGACTCTGCAATAA
S100A7	F	TGCTGACGATGATGAAGGAG
	R	ATGTCTCCCAGCAAGGACAG
S100A8	F	GGGAATTTCCATGCCGTCT
	R	CCTTTTTCCTGATATACTGAGGAC
S100A9	F	CAGCTGGAACGCAACATAGA
	R	TCAGCTGCTTGTCTGCATTT
S100A12	F	AGCATCTGGAGGGAATTGTCA
	R	GCAATGGCTACCAGGGATATGAA

## Data Availability

All data used to support the findings of this study are included within the article.
